# Multimodality Imaging in Secondary Mitral Regurgitation

**DOI:** 10.3389/fcvm.2020.546279

**Published:** 2020-12-22

**Authors:** Harish Sharma, Boyang Liu, Hani Mahmoud-Elsayed, Saul G. Myerson, Richard P. Steeds

**Affiliations:** ^1^Institute of Cardiovascular Sciences, University of Birmingham, Birmingham, United Kingdom; ^2^Department of Cardiology, Queen Elizabeth Hospital, University Hospitals Birmingham National Health Service (NHS) Foundation Trust, Birmingham, United Kingdom; ^3^Department of Cardiology, Al-Nas Hospital, Cairo, Egypt; ^4^Department of Cardiovascular Medicine, University of Oxford, Oxford, United Kingdom

**Keywords:** mitral regurgitation, echocardiogaphy, magnetic resonanace imaging, computed tomography, ischaemic heart disease

## Abstract

Secondary mitral regurgitation (sMR) is characterized by left ventricular (LV) dilatation or dysfunction, resulting in failure of mitral leaflet coaptation. sMR complicates up to 35% of ischaemic cardiomyopathies (1) and 57% of dilated cardiomyopathies (2). Due to the prevalence of coronary artery disease worldwide, ischaemic cardiomyopathy is the most frequently encountered cause of sMR in clinical practice. Although mortality from cardiovascular disease has gradually fallen in Western countries, severe sMR remains an independent predictor of mortality (3) and hospitalization for heart failure (4). The presence of even mild sMR following acute MI reduces long-term survival free of major adverse events (1). Such adverse outcomes worsen as the severity of sMR increases, due to a cycle in which LV remodeling begets sMR and vice versa. Current guidelines do not recommend invasive treatment of the sMR alone as a first-line approach, due to the paucity of evidence supporting improvement in clinical outcomes. Furthermore, a lack of international consensus on the thresholds that define severe sMR has resulted in confusion amongst clinicians determining whether intervention is warranted (5, 6). The recent Cardiovascular Outcomes Assessment of the MitraClip Percutaneous Therapy for Heart Failure Patients with Functional Mitral Regurgitation (COAPT) trial (7) assessing the effectiveness of transcatheter mitral valve repair is the first study to demonstrate mortality benefit from correction of sMR and has reignited interest in identifying patients who would benefit from mitral valve intervention. Multimodality imaging, including echocardiography and cardiovascular magnetic resonance (CMR), plays a key role in helping to diagnose, quantify, monitor, and risk stratify patients for surgical and transcatheter mitral valve interventions.

## Introduction

Mitral regurgitation (MR) is the most common valvular disease, and prevalence is increasing with improved life expectancy in the Western world (8). Primary MR (pMR) is due to a structural or degenerative disorder of the valve apparatus with left ventricular (LV) impairment or dilatation resulting from the regurgitation. Conversely in secondary MR (sMR), the mitral apparatus is relatively normal and failure of leaflet coaptation is directly attributable to a diseased LV which is subject to contractile impairment and/or pathogenic dilatation. sMR is observed in up to 35% of patients with ischaemic cardiomyopathy (ICM) due to post-infarct LV remodeling (1, 9) and 57% of patients with dilated cardiomyopathy (DCM) (2). Although sMR occurs more frequently in patients with DCM, ICM is the most prevalent form of cardiovascular disease worldwide and therefore is the most common cause of sMR. The presence of even mild sMR adversely impacts prognosis from time of onset (1, 2). Therefore, early diagnosis, careful monitoring and timely intervention are critical. The aim of this article is to outline the advantages and disadvantages of echocardiography, cardiovascular magnetic resonance (CMR) and computer tomography (CT) in the management of sMR.

## Pathophysiology of sMR

**Figure 1:**
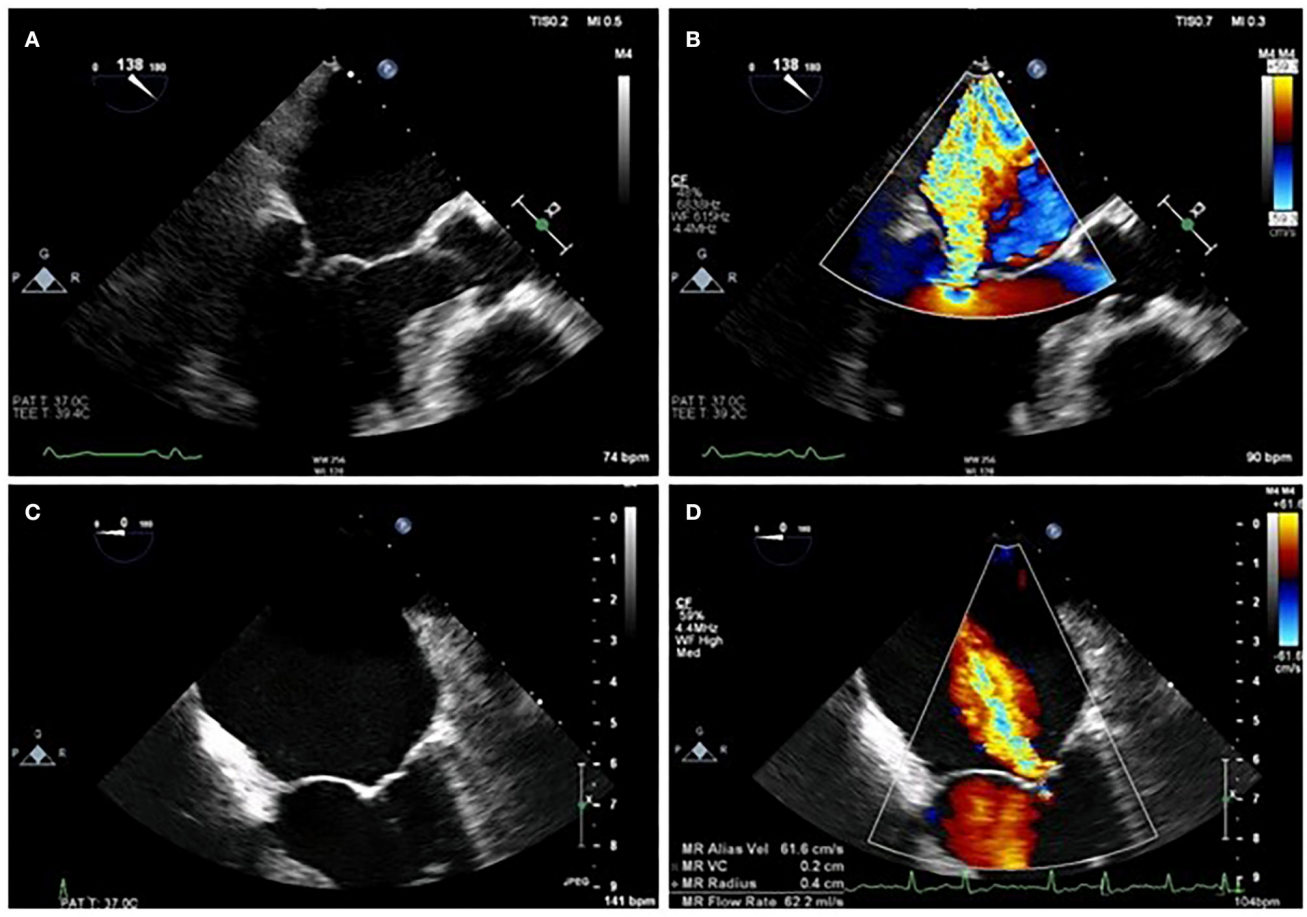
Difference between asymmetrical **(A,B)** and symmetrical **(C,D)** LV remodeling on MR jet direction on 2D TOE.

Normal function of the mitral valve (MV) relies on leaflet coaptation during ventricular systole. This requires undistorted LV geometry, intact MV apparatus, synchronous contraction of papillary muscles and a balance between opening and closing forces generated by LV contraction and relaxation. Failure of any of these processes results in sMR, although the mechanism depends on the insult to the LV. For example, an inferolateral myocardial infarction may cause focal myocardial remodeling and an akinetic superolateral papillary muscle, leading to asymmetric chordal traction on posterior and anterior leaflets, apical displacement of the coaptation point and ultimately eccentric, posteriorly directed MR (Carpentier type IIIb mechanism). Less frequently, symmetric dilatation of the LV occurs due to dilated cardiomyopathy (DCM) or severe 3-vessel coronary disease, leading to symmetric annular dilatation, equal papillary muscle displacement, and tenting of both leaflets resulting in a central jet of regurgitation (Carpentier type Ia mechanism) (Figure 1).

**Figure 2:**
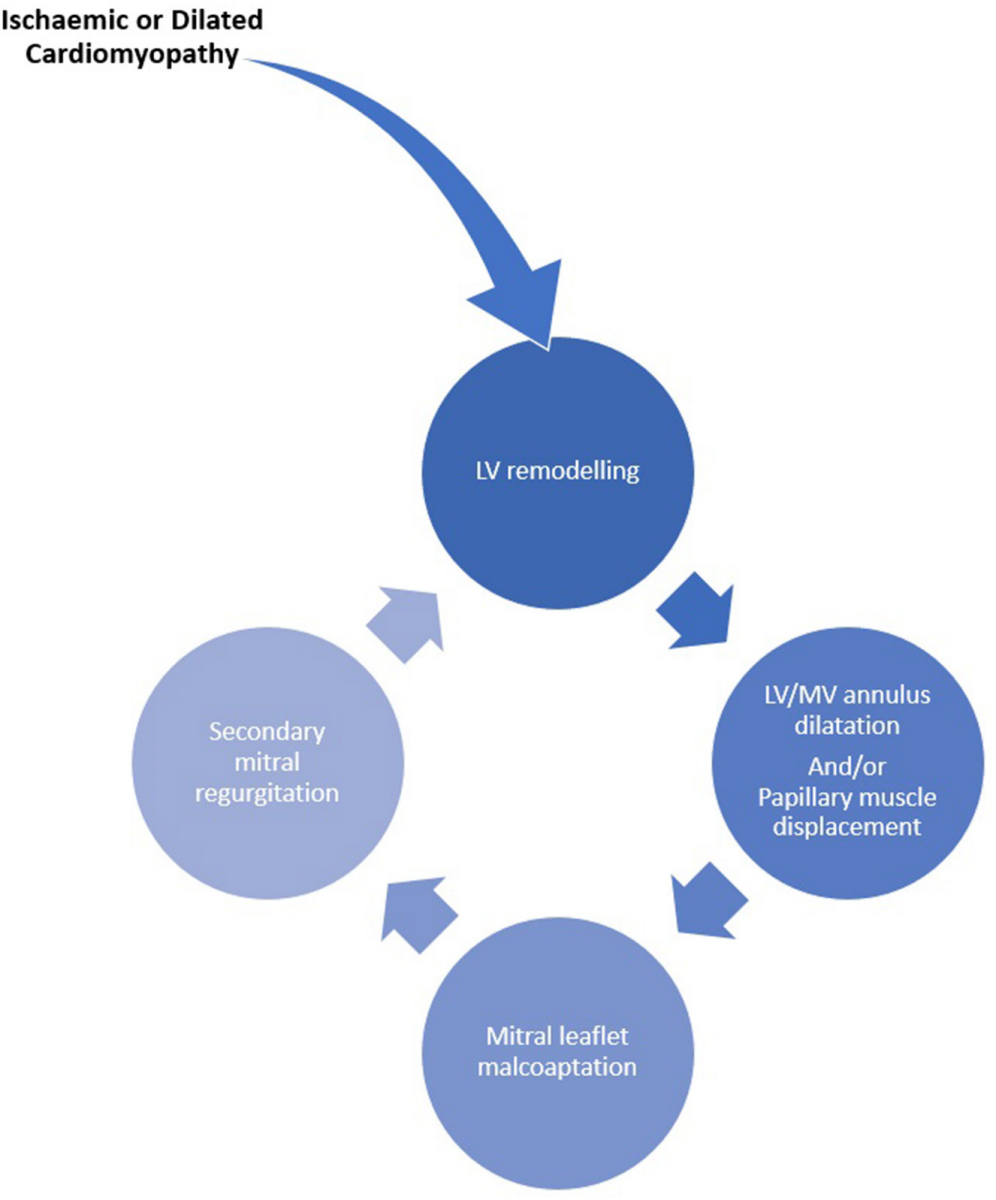
Relationship of LV remodeling with sMR.

A vicious cycle ensues in which the resultant sMR causes left atrial and ventricular volume overload and dilatation, which in turn promotes further sMR (Figure 2). Chronic severe sMR results in compensatory left atrial (LA) and ventricular dilatation and eventual dysfunction. Over time, reduced chamber compliance results in pulmonary venous hypertension and ultimately right ventricular pressure overload.

## Diagnosis and Identifying Etiology

Reliance on clinical examination to detect sMR lacks sensitivity, and the main diagnostic tool is transthoracic echocardiography (TTE) with color flow doppler. On finding MR, the role of the imager is to determine the mechanism of MR, through the assessment of the mitral leaflets, annulus, chordae and papillary muscles, along with associated changes in the ventricle and atrium.

### Imaging the Mitral Leaflets

**Figure 3:**
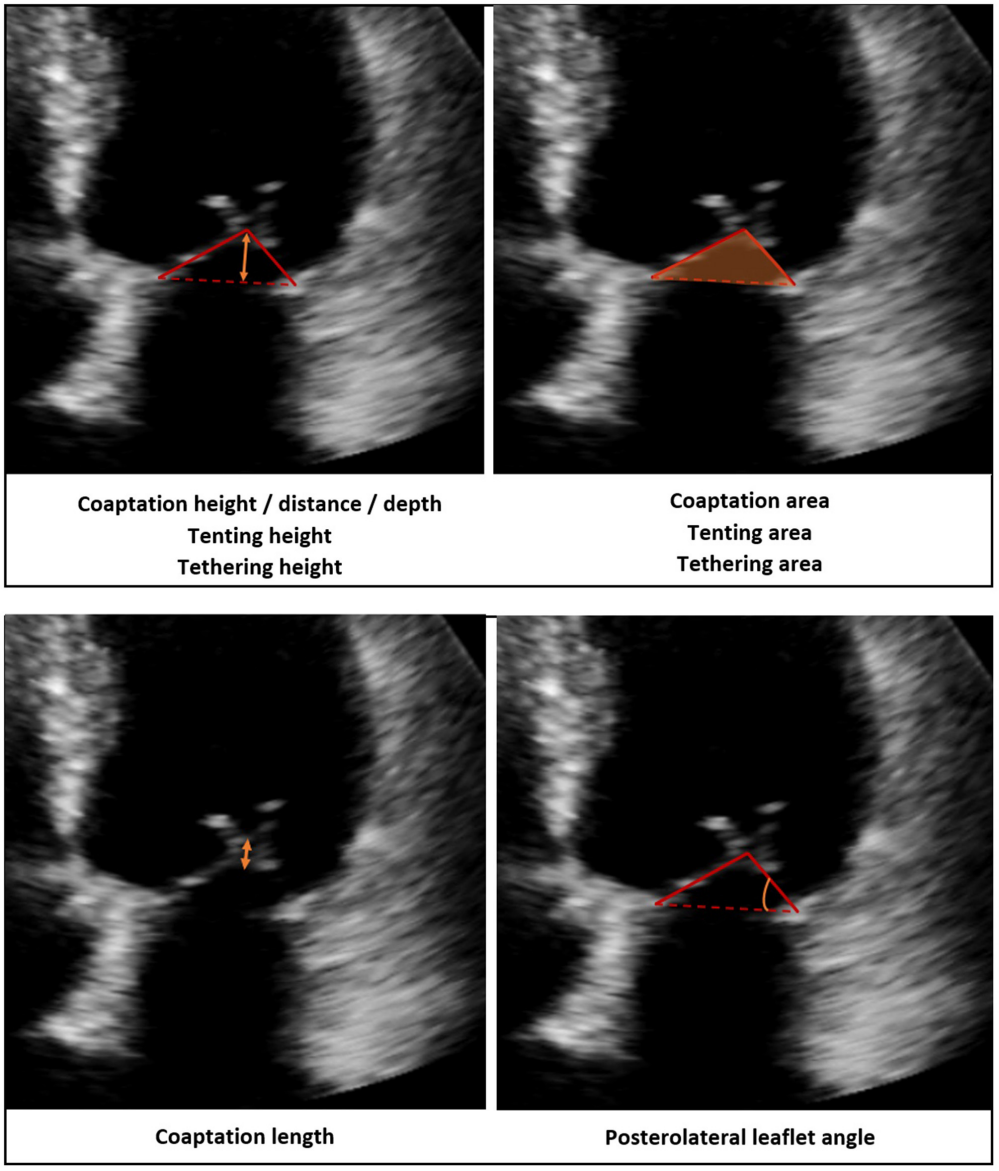
Measurements of leaflet geometry by 2D TTE.

Although the classical definition of sMR requires that the MV leaflets are normal, it is now known that changes occur in response to the stresses exerted upon the tented leaflets that counteract the loss of leaflet coaptation. These compensatory changes include progressive leaflet lengthening, thickening and fibrosis (10, 11). 2D TTE using fundamental imaging is the main modality for anatomic assessment of leaflet thickness (normal or increased), motion (normal, restricted or increased) and presence and distribution of calcification (none, mild, moderate, severe). 3D TTE adds incremental value in providing a comprehensive assessment of the entire valve, including the ability to identify the specific scallop(s) involved. 3D TOE however, is the gold-standard method for full assessment of the valve and scallops compared to surgical findings, and offers dynamic quantification of leaflet geometry throughout the cardiac cycle (12). Leaflet configuration can influence likelihood of successful surgical repair. The following configurations are shown to be predictors of failure of repair: Leaflet coaptation distance > 1 cm, tenting height > 6 mm, tenting area > 2.5 cm^2^ on TTE and > 1.6 cm^2^ on TOE and posterior leaflet angle > 45°. A full summary of these factors are categorized in Table 1. If these factors are present, this may influence the decision re: mitral valve repair vs. replacement or whether intervention should proceed at all (Figure 3).

**Table 1:** Pre-operative predictors of recurrence of sMR following surgical mitral valve repair.

**Pre-operative predictors of failure of surgical mitral valve repair in sMR**			
	**Modality**	**Accuracy**	**Reference**
**MITRAL VALVE**			
MR grade > 3.5	TTE	Sensitivity 42% Specificity 81%	(19)
Central or complex regurgitant jet	TTE	N/A	(20)
Leaflet coaptation distance > 1 cm	TTE	Sensitivity 64% Specificity > 90%	(21)
Leaflet tenting height > 6 mm	TTE	N/A	(22)
Leaflet tenting area > 2.5 cm^2^	TTE	Sensitivity 64% Specificity > 90%	(21)
Posterior leaflet angle > 45°	TTE	Sensitivity 100% Specificity 97% PPV 92% NPV 100%	(21)
P3 tethering angle < 29.9°	Intraoperative 3D TOE	AUC 0.92 (0.84–1.00)	(23)
Leaflet tenting area > 1.6 cm^2^	Intraoperative TOE	Sensitivity 80% Specificity 54%	(19)
Mitral Annular Diameter > 3.7 cm (long-axis view in mid-systole)	Intraoperative TOE	Sensitivity 84%, Specificity 76%	(19)
**LEFT VENTRICLE**			
EDD > 6.5 cm	TTE	N/A	(21, 22, 24)
ESD > 5.1 cm	TTE	N/A	(21)
ESV > 145 ml	TTE	Sensitivity 90% Specificity 90%	(25)
Interpapillary muscle distance > 2 cm	TTE	Sensitivity 96% Specificity 97%	(26)
Posterior papillary-fibrosa distance > 4 cm	TTE	N/A	(27)
Wall motion score index > 1.5	TTE	Sensitivity 80% Specificity 82%	(25)
Systolic sphericity index > 0.7	TTE	Sensitivity 100% Specificity 100%	(25)
Myocardial performance index > 0.9	TTE	Sensitivity 85% Specificity 84%	(25)
Basal aneurysm/dyskinesis	TTE	N/A	(28)
Non-viable LV segments > 3	CMR	N/A	(29)
Non-viable posterior wall LV segment(s)	CMR	N/A	(29)

*MR, Mitral Regurgitation; TTE, Transthoracic Echocardiogram; TOE, Transoesophageal Echocardiogram; CMR, Cardiovascular Magnetic Resonance; EDD, End-Diastolic Diameter; ESD, End-Systolic Diameter; ESV, End Systolic Volume*.

In patients with poor acoustic windows or who are unable to tolerate TOE, CMR and ECG-gated CT both offer good alternatives for visualizing leaflet anatomy and quantitative assessment of MR, but lack the 3D views that are extremely helpful for the dynamic assessment of scallops. Nevertheless, CT provides useful measurements in sMR that correlate with 3D echo including open mitral valve leaflet area (*R*^2^ = 0.97; *P* < 001), closed mitral leaflet area (*R*^2^ = 0.84; *P* < 0.01), tenting area and volume (*R*^2^ = 0.84), and papillary muscle tethering distances (superolateral *R*^2^ = 0.90; inferoseptal *R*^2^ = 0.84) (30). CT is also the best method to localize and quantify calcification in and around the MV apparatus, a role which is likely to grow with the use of transcatheter mitral valve repair (TMVR). CMR has lower temporal resolution compared to echocardiography but has high spatial resolution that provides a good alternative modality for defining leaflet anatomy, including individual scallops (31), which compares well to 2D TOE (32).

### Imaging the Mitral Annulus

**Figure 4:**
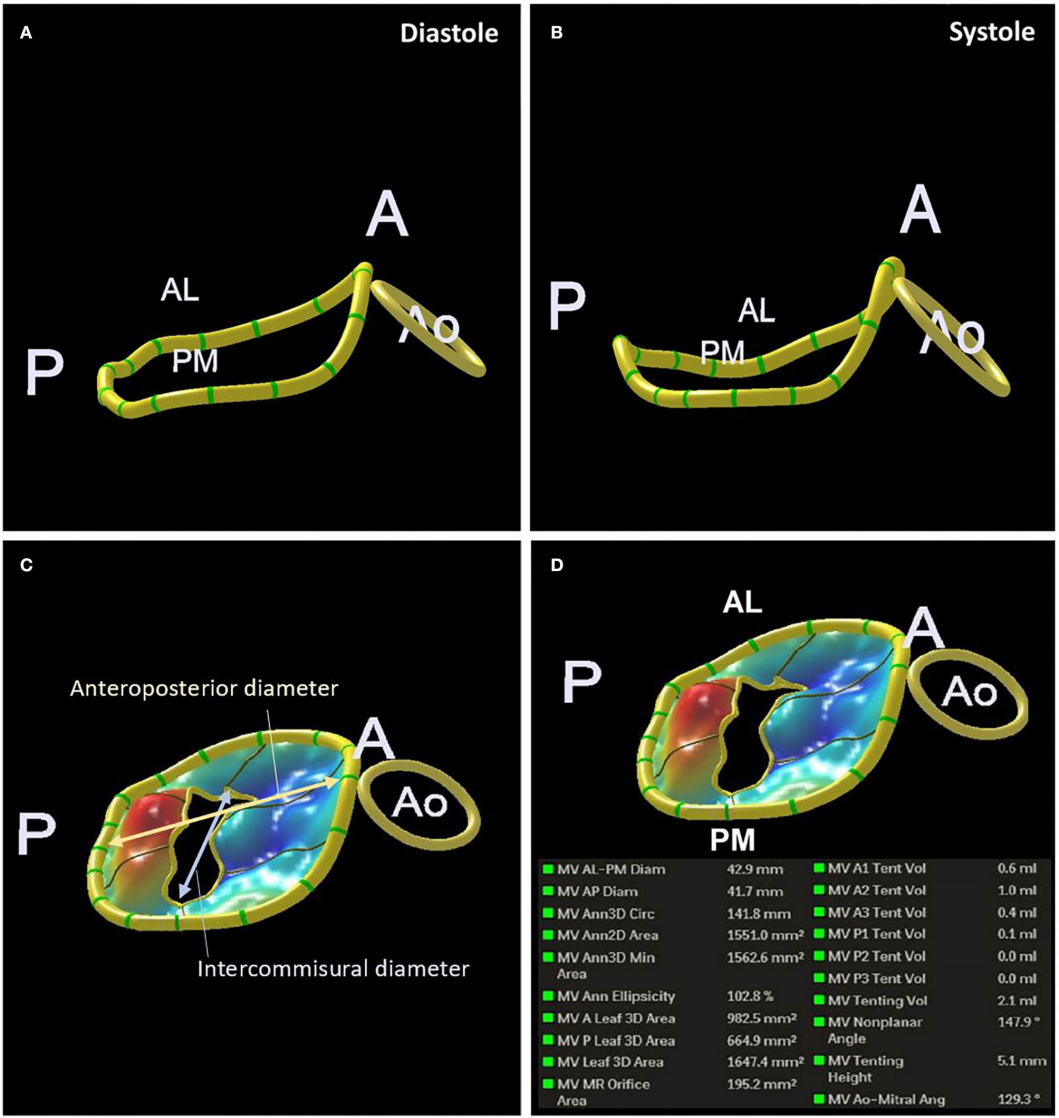
3D TOE reconstruction of the mitral annulus in systole **(A)** and diastole **(B)**. Measurements of anteroposterior and Intercommisural diameters **(C)**. An example of the measurements of the mitral annulus obtained **(D)**.

The normal mitral annulus is elliptical shaped in cross-section and saddle-shaped in 3 dimensions. The highest points are near the superolateral commissure while the lowest points are located at the base of P2 and P3 (33). The annulus changes configuration throughout the cardiac cycle to maintain leaflet coaptation, from D-shaped in mid-systole to a more circular shape in early diastole, reducing annular area by 20–30% across the cardiac cycle. The annulus also moves away from the apex of the heart in diastole, although motion of the posterior aspect is more marked than the anterior annulus, and there is conformational change with accentuation of the saddle shape in systole. All of these movements are affected by ischaemia, which tends to reduce the dynamic change in area, impair longitudinal motion, and flatten the annulus, leading to increased leaflet stress (34–37). The extent of remodeling is greater following anterior compared to inferior myocardial infarction (MI) (38). These changes can be tracked by high spatial and temporal resolution imaging using 3D TOE. Figure 4 is an example of a 3D reconstructed model at end-diastole and end-systole, although annular parameters can be tracked for each frame acquired through the cardiac cycle. 2D measurements, whether by TTE or TOE, are only an approximation, since accurate alignment to the major axes is only possible with multi-planar imaging. Using the parasternal long axis view on 2D TTE (or left ventricular outflow tract view on CMR), the mitral annulus is considered dilated if the diastolic ratio of annulus/anterior leaflet is > 1.3, or if the absolute annulus diameter is > 3.5 cm (27). The upper reference limit of normal for indexed mitral annulus area by 3D TOE is 6.9 cm^2^/m^2^ (39). While 3D TTE and TOE are gold standard, there is increasing evidence supporting the use of ECG-synchronized 3D multi-detector CT in the planning of TMVR, particularly for assessing annulus dimensions, landing zones, alignment with the inter-commissural lines for coaxial deployment and prevention of LVOT obstruction (40).

### Imaging the Subvalvular Apparatus

**Figure 5:**
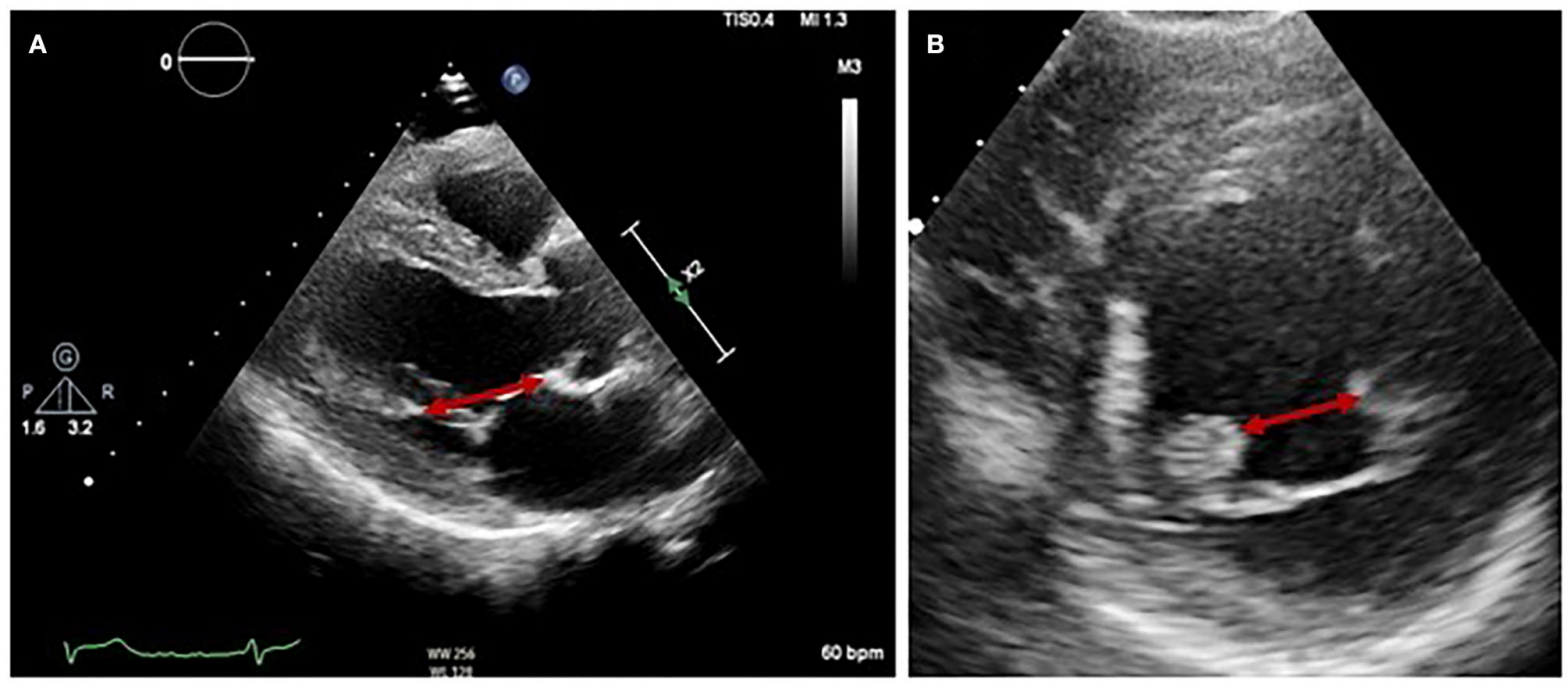
2D TTE demonstrating papillary-fibrosa distance **(A)** & interpapillay distance **(B)**.

Normal functioning of the subvalvular apparatus is intimately linked to the LV and can have a profound impact on the severity of sMR. There is a higher incidence of sMR in patients with inferior than anterior myocardial infarction (MI) despite less severe LV remodeling with the former. This is due to localized inferobasal LV remodeling following inferior MI, resulting in displacement of the contiguous papillary muscle (38, 41, 42). Note should be drawn to recent autopsy data supporting a change in terminology in relation to the papillary muscles, from posteromedial to inferoseptal, and anterolateral to superolateral (43, 44). The extent of papillary muscle displacement is best evaluated on 3D TOE or CMR. The most important measurements include the interpapillary distance and posterior papillary-fibrosa distance. Interpapillary muscle distance >2 cm and posterior papillary-fibrosa distance >4 cm are considered to be unfavorable characteristics for MV repair (Figure 5) (27).

Direct papillary muscle infarction (PMI) also contributes to sMR in up to a third of patients following acute MI. Late gadolinium enhancement (LGE) of the papillary muscles on CMR can diagnose PMI but the prognostic significance of these findings are unclear (45, 46). Following 1–3% of acute MI, infarctions of papillary muscles or chordae tendineae can lead to rupture, leading to flail mitral leaflets and severe valvular incompetence (47). If left untreated, in-hospital mortality approaches 80% (48). TTE is often used in the acute setting to confirm clinical suspicion of acute severe MR but TOE is usually required to elucidate the mechanism, due to the superior imaging of the subvalvular apparatus.

### Imaging the Left Ventricle

The presence of sMR increases the poor prognosis of reduced ventricular function, and the frequency and severity of adverse outcomes increase as the MR worsens. Heart failure affects the majority of patients with sMR within 5 years, including those who have normal LVEF (49). The interaction between sMR and LV size and function is therefore a critical component of multi-modality imaging assessment; the purpose of which, is to define anatomy and pathophysiology of the MR, determine prognosis and select patients suitable for intervention.

Transthoracic echocardiography (TTE) is usually the first line investigation for the investigation of LV size and function by 2D Simpson's biplane method. 3D volumetric assessment is more accurate and reproducible (50) but under-estimates volumes, even with the use of contrast, compared to the gold-standard CMR methods (51–53). In severe compensated sMR, LV systolic function is hyperdynamic due to the Frank-Starling mechanism. This can give a false impression of the true impairment of LVEF. Measurement of global longitudinal strain (GLS) with speckle tracking provides a less load-dependent method of assessing LV function, but is not currently routinely acquired. Greater impairment of GLS is associated with higher mortality and is an independent predictor of long-term outcome in patients with chronic ischaemic cardiomyopathy (50). Furthermore, in a study of 650 patients with severe sMR, GLS was shown to be an independent predictor of all-cause mortality, while LVEF was not (54). Stress echocardiography provides additional information where there is a discrepancy between patient symptoms and resting severity of MR by detecting those in whom sMR worsens with exercise. In such patients, a blunted LVEF response to exercise (mean increase of 5 vs. 10%) and an effective regurgitant orifice area (EROA) increase > 0.13 cm^2^ are independent predictors of pulmonary oedema (55) and cardiac mortality (15), respectively.

CMR is not only the gold standard for measuring LV structure (EDV, mass/volume ratio) and function (LVEF; ESV) but also quantifies pathophysiological changes in the myocardium. CMR measures the presence, extent and location of post-infarction scar using LGE and thereby identifies those with greater risk of sMR (56) and higher mortality in those undergoing surgery (3). Furthermore, CMR can quantify diffuse interstitial fibrosis in the form of extracellular volume (ECV) using T1 mapping. In sMR, ECV expansion may occur secondary to the MR or the underlying LV pathology. Although increased T1 and ECV are important predictors of outcome in IHD (57), with incremental predictive value to LVEF and LGE (56), to date, there are no data on the relative importance of ECV expansion vs. volume overload in sMR, nor on the ability of ECV to predict outcomes in sMR.

### Summary Recommendations

**Figure 6:**
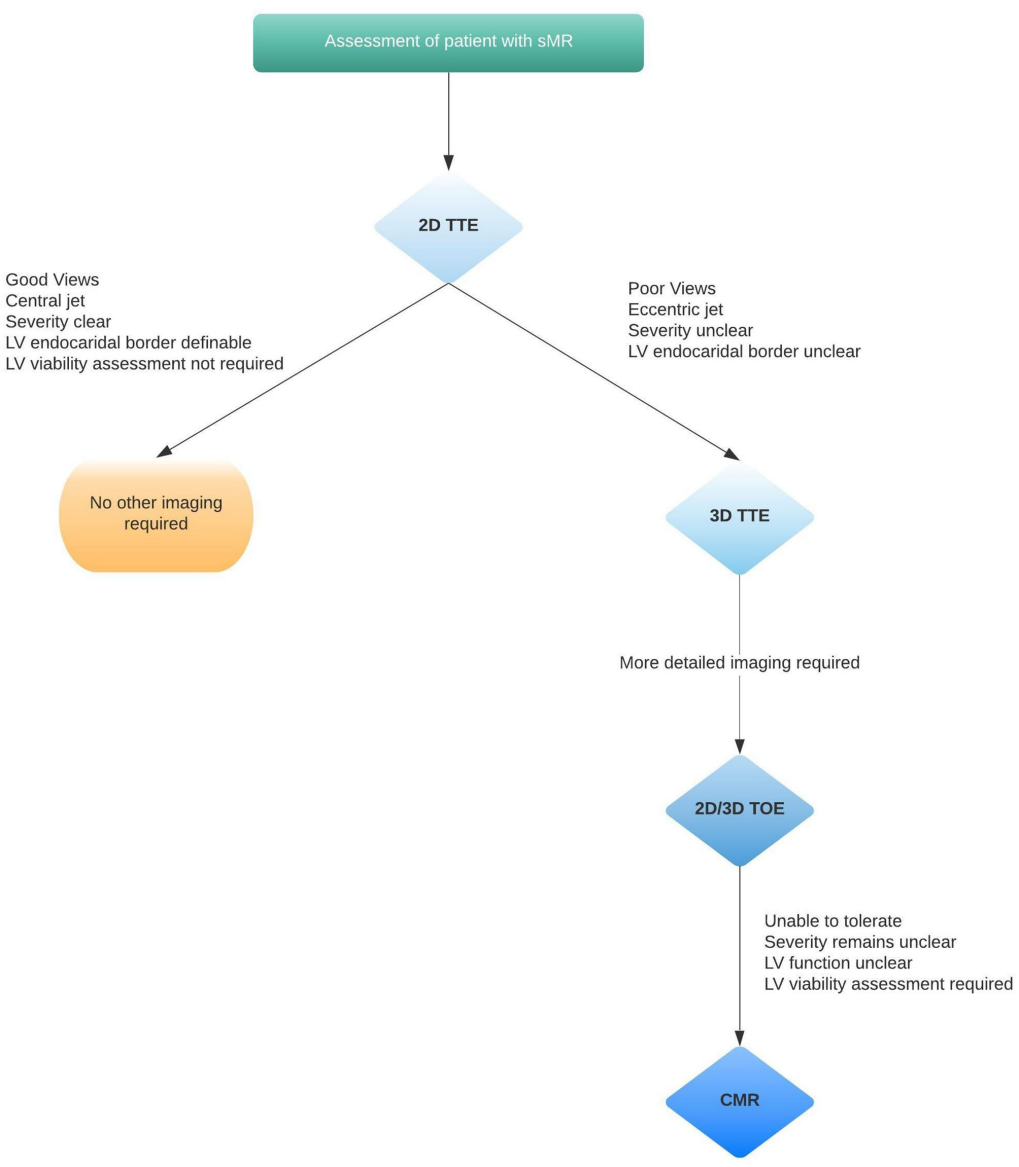
Clinical integration of the different imaging modalities.

- 2D and 3D assessment by TTE supplemented by TOE are the investigations of choice for imaging the pathognomonic changes to the mitral leaflets, annulus, chordae and papillary muscles in ischaemic sMR.
- CMR or CT assessment of the valve is useful if adequate imaging is not obtained with echocardiography (Figure 6).
- LV volumetric assessment by 3D echo is superior to 2D echo, however, CMR is the gold-standard technique to assess the LV due to superior spatial resolution, allowing accurate measurement of LV volume, geometry, fibrosis and extent of scar.

## Quantifying Severity of sMR

### Role of Echocardiography

Current European and American guidelines recommend an integrated approach to the assessment of sMR, in part recognizing the limitations of individual qualitative, semi-quantitative, and quantitative echocardiographic parameters and in part understanding that not every measurement can be made in all patients (5, 58). Although TTE is the first-line investigation for sMR (27), quantifying severity is much more difficult than in primary MR for several reasons. Firstly, LV stroke volume is often reduced by the impact of IHD, which means that the RVol in sMR is often smaller than in pMR. There is disagreement between European and US guidelines on the size of RVol in severe sMR, although evidence supports an adverse prognostic outcome at an RVol >30 ml (18). Secondly, the shape of the regurgitant orifice is often altered in sMR from a hemisphere to an ellipse, meaning that quantification using measures such as vena contracta width (VCW) and flow convergence is not as reliable as in primary MR. These individual measures do however retain diagnostic and prognostic utility Table 2. For example, in a study of 356 patients with severe sMR, VCW > 0.4 cm on TTE predicted the combined endpoint of death, HF hospitalization and treatment (16). Thirdly, haemodynamic status, LV end-diastolic pressure and left atrial pressure may be profoundly altered by the effects of IHD, which in turn can all alter measures of sMR severity, independent of the size of the regurgitant orifice. For example, although a jet of sMR on color Doppler > 4.5 cm^2^ or > 50% of the LA area is associated with increased mortality at 1 year (OR 4.47 cf mild or absent MR) (14), this is not a recommended way of assessing severity of regurgitation because jet area is so dependent on haemodynamic status. Likewise, blunting or reversal of the S wave on pulmonary venous flow pattern on pulse Doppler in sMR may occur simply due to high LVEDP and LA pressure, independent of the severity of MR. For example in a study of 119 patients with ischaemic or dilated cardiomyopathy, 48 (40%) patients had S wave blunting without severe MR and 24 (20%) had no MR at all (59).

**Table 2:** sMR assessed by qualitative and quantitative echocardiographic methods.

	**Number of patients (% ischaemic)**	**Cut off values**	**Predicted Outcome**	**Reference**
**QUALITATIVE**				
Size of jet on color flow doppler	272 severe sMR (100%)	>50% left atrial area	Mean survival 676 ± 41 days RR of mortality 1.84 (1.43–2.38)	(13)
	35 moderate-severe (58%)	Jet area > 4.5 cm^2^	1-year mortality 51% OR of 1-year mortality 4.47 vs. mild/absent MR	(14)
**QUANTITATIVE**				
MV E wave decel time VCW (by TTE)	98 sMR patients (100%) 336 severe sMR (57%)	Survivors 182 msec vs. Non-survivors 148 msec > 0.4 cm	Independent predictor of mortality Combined end point of death, HF hospitalization and transplant	(15) (16)
VCA (by 3D TOE)	259 moderate/ severe sMR (44%)	> 0.39 cm^2^	Detection of severe sMR: Sensitivity 92%; Specificity 90%	(17)
				
EROA (by 3D TOE PISA)	259 moderate/ severe sMR (44%)	> 0.24 cm^2^	Detection of severe sMR: Sensitivity 83%; Specificity 74%	(17)
EROA (by TTE PISA)	194 severe sMR (100%)	> 0.20 cm^2^	Adjusted RR of mortality 2.23 (1.31–3.79)	(18)
EROA (by TTE PISA or doppler)	98 severe sMR (100%)	0.2 cm^2^ at rest > 0.13 cm^2^ on exertion	Independent predictors of mortality	(15)
RVol (by EROA/Doppler)	194 (100%)	> 30 ml	Adjusted RR of mortality 2.05 (1.30–3.23)	(18)

*sMR, Secondary MR; MV, Mitral Valve; HF, Heart Failure; VCW, Vena Contract Width; VCA, Vena Contracta Area; EROA, Effective Regurgitant Orifice Area; TTE, Transthoracic Echocardiogram; TOE, Transoesophageal echocardiogram; RVol, Regurgitant Volume; RR, Risk Reduction; MR, Mitral Regurgitation*.

The limitations of 2D echocardiographic assessment can be mitigated by using an algorithm incorporating multiple parameters to risk stratify patients and identify those in need of valvular intervention. In a study of 423 patients wth chronic heart failure, patients with EROA < 0.2 cm^2^, RVol < 30 ml were identified as having low mortality risk while those with EROA > 0.3 cm^2^ and Rvol > 45 ml were high risk. For the intermediate group (EROA 0.2–0.29 cm^2^ and RVol 30–44 ml), a regurgitant fraction above and below 50% was found to be a threshold which could divide patients into the high and low risk categories, respectively (60).

**Figure 7:**
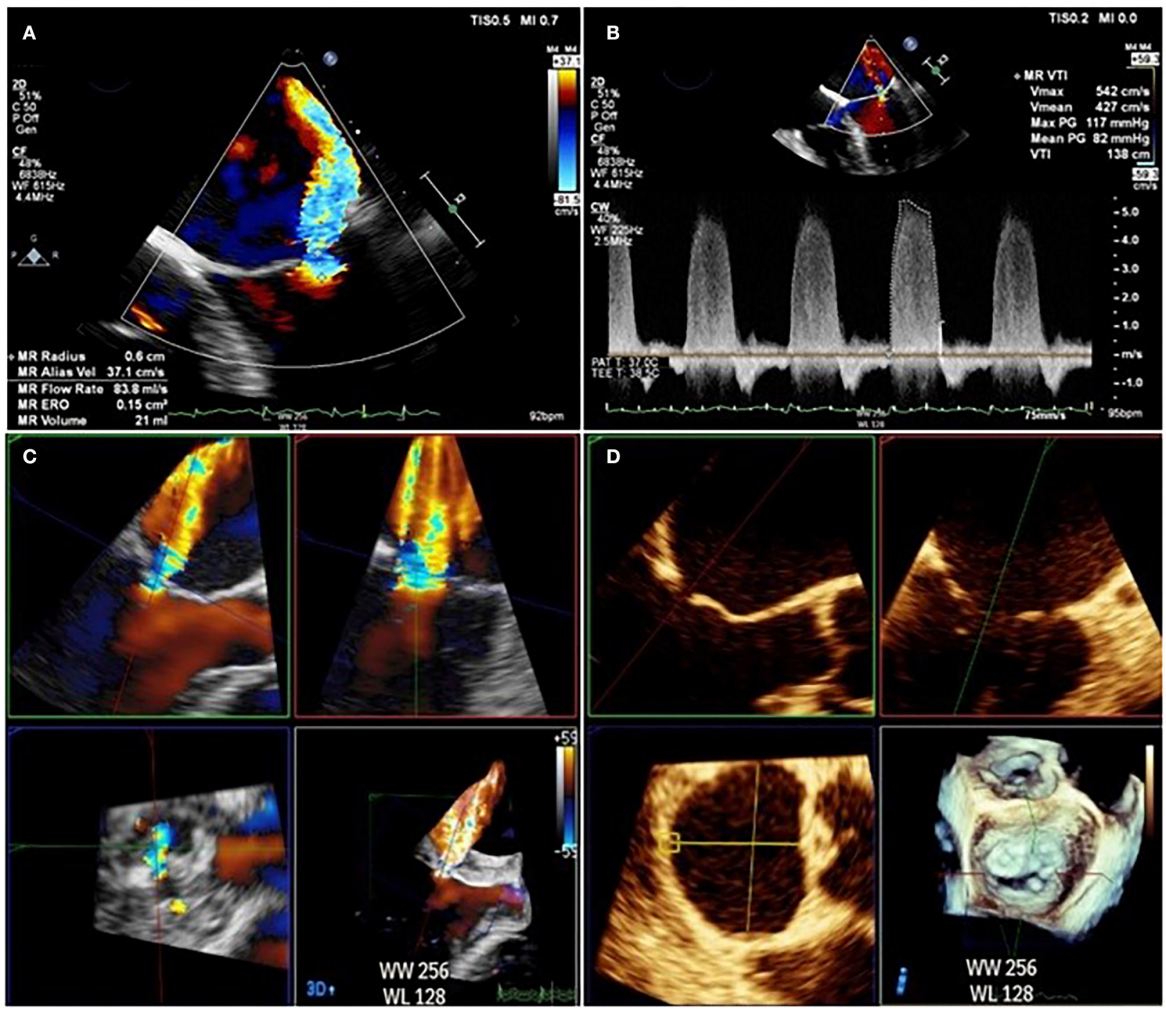
Difference between EROA derived from 2D TOE **(A,B)** calculated at 0.15 cm^2^ and EROA derived from 3D TOE **(C,D)** in the same patient calculated at 0.45 cm^2^.

The limitations of 2D echocardiography can also be overcome by routine use of 3D color Doppler with multiplanar reconstruction (MPR). The problem of non-hemispherical shape can be surmounted by using biplane to measure VCW in perpendicular views or by using multiplanar reconstruction to measure vena contracta area (VCA). While the threshold for VCA severity remains to be clearly defined, 98% of sMR patients with an EROA > 0.2 cm^2^ have a VCA of > 0.4 cm^2^, a figure which correlates closely with other measures of severe regurgitation (17). 3D color Doppler can be used to directly measure the EROA, and has been validated against other measures on echocardiography and externally validated against phase-encoded CMR (61). 3D values for EROA tend to be larger than those calculated using the 2D color Doppler PISA method, as demonstrated in Figure 7. The clinical impact of this under-estimation using the 2D color Doppler method was emphasized in a study of 111 pMR and 100 sMR patients, in which a third of those patients with severe MR on 3D were mis-classified as having non-severe MR by 2D EROA (62).

### Role of CMR

**Figure 8:**
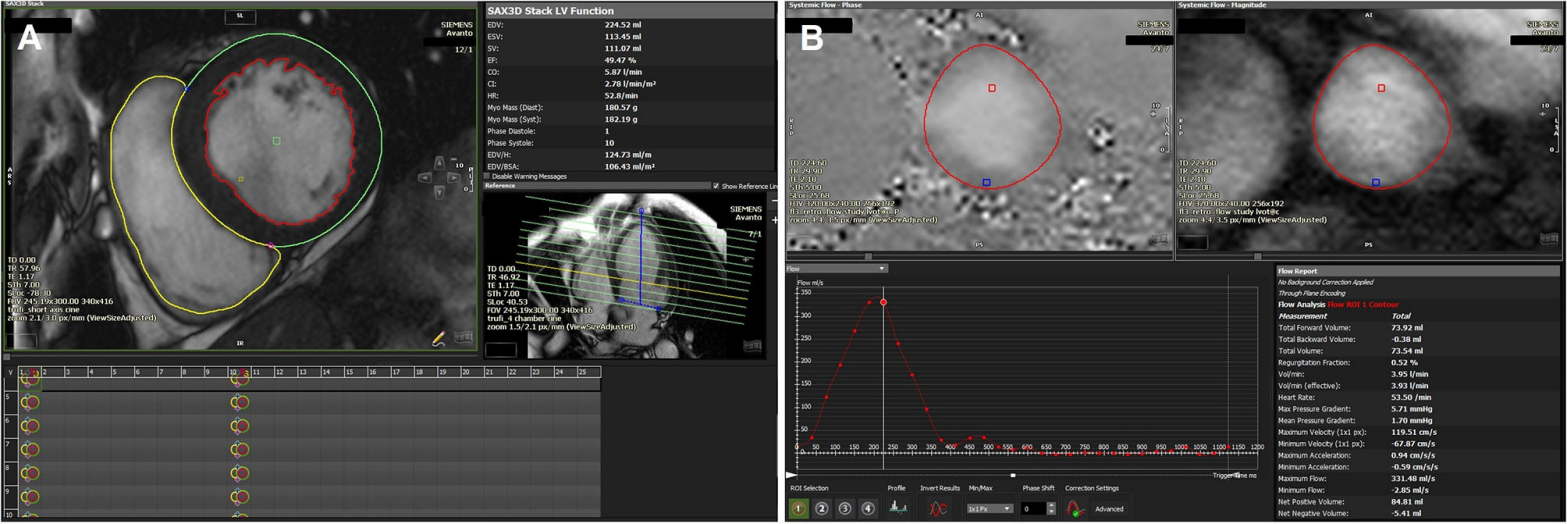
**(A)** Volumetric assessment of LV/RV short axis stack using the detailed contouring method showing a dilated LV (LV volume 106 ml/m^2^) with mildly impaired LVEF (49%). Calculated LV stroke volume (SV): 111 ml, RV SV: 74 ml. **(B)** Phase contrast aortic flow analysis showing a total forward volume of 74 ml with no significant regurgitation. Estimated MR volume based on RV/LV SV variation and volumes-derived SV and phase contrast aortic flow: 111–74 = 37 ml.

RVol is most often calculated in CMR by measuring the difference between LV stroke volume (from a stack of short axis steady state free precession cine images) and aortic stroke volume from phase-contrast velocity flow mapping in the aortic sinus (Figure 8) (63). As internal validation, aortic stroke volume should be within 5% of the pulmonary artery stroke volume in the absence of other regurgitant valves or intracardiac shunts. The advantage of CMR is that the calculated RVol is independent of the number, shape or eccentricity of the regurgitant jet(s), their duration through systole, and CMR requires no contrast agents or ionizing radiation. The disadvantages are that phase-contrast mapping can be affected by phase offset error, while irregular cardiac rhythm, poor breath-holding, and basal slice selection reduce the reliability of calculated LV stroke volume. There is however, evidence that quantifying MR by CMR improves reproducibility and may be a better arbiter of outcome than echocardiography (64). Firstly, disagreement in the grading of MR severity by echo and CMR is common. Of the 5 studies comparing CMR to 2D echo, there was moderate to good agreement (*r* = 0.6–0.92) in only 26–66% patients when categorizing MR as severe (65–69). Of the 4 studies comparing CMR to 3D echo, agreement improved to 49–79% (62), but there was considerable variation in results across studies (70–72). Secondly, reproducibility in all studies is consistently better using CMR than echo, whether by 2D or 3D (64). Thirdly, grading by CMR has the potential to be a better predictor of patient outcome, although data that shows improved prediction of LV reverse remodeling (65) and of need for surgery and mortality are limited to pMR only (73). More data are needed in sMR, not only to validate markers of severity of sMR against outcomes but also to define the potential role of tissue characterisation of the LV in predicting outcome from intervention.

### Summary Recommendations

Multiparametric TTE is the mainstay for quantifying sMR;

- There are major limitations of 2D TTE and TOE quantification of sMR;
- 3D echo overcomes many of the limitations of 2D but reproducibility is an issue;
- Quantification of sMR by CMR is more reproducible and may be a better arbiter of outcome but further research is needed.

## Imaging to Facilitate Intervention

Multimodality imaging plays a central role in predicting benefit from intervention, including accurate quantification of sMR relative to the degree of LV remodeling, and identification of mitral valve characteristics that are more amenable to successful intervention.

### Mitral Valve Surgery

Although mitral annuloplasty produces excellent long-term results in pMR (74), this is not the case in sMR, where only 75% of patients are free of recurrent MR at 2 years (29). Trials of surgical intervention for sMR have generally demonstrated poor results, despite revascularisation and correction of the MR. Concomitant MV surgery at the time of CABG did not confer a survival advantage compared to CABG alone (75, 76). Patients with severe sMR who were randomized to valve repair or replacement had similar 2-year cumulative mortality rates of 19 and 23%, respectively, with high rates of recurrent MR in those undergoing repair (77). In patients with moderate sMR, surgical correction at the time of CABG did not improve LV remodeling, adverse event rates, hospitalization, or survival (78). In contrast, the RIME study (the only study using CMR for pre-operative assessment) demonstrated that although surgical correction of sMR did not improve survival, operated patients had improved functional capacity and LV reverse remodeling (79).

Pre-operative characteristics that are associated with increased failure rates on echo include more extreme LV remodeling (LV end-diastolic diameter > 65 mm) and greater distortion of the MV apparatus (leaflet tenting height > 6 mm) (22). Similar conclusions were drawn in a prospective study using 3D TOE and CMR, again identifying a combination of more extreme LV remodeling (indexed LVESV > 45.4 ml/m^2^) and greater tethering (assessed by measurement of the posterior leaflet angle > 40.2°) as strongly associated with high likelihood of sMR recurrence (29). The potential added value of CMR tissue characterisation was demonstrated by the finding that sMR recurrence was more likely in those with more extensive infarction (> 3 non-viable LV segments or > 1 non-viable segment in the posterior wall) (29). A full list of imaging predictors of sMR recurrence following surgical annuloplasty are listed in Table 1.

### Transcatheter Repair

While a number of devices are in development, transcatheter edge-to-edge repair using MitraClip is currently the most widely used device. Other devices such as Cardioband and the Carillon mitral contour system have CE approval and have shown promising results in reducing mitral regurgitant volume (80, 81). In the Endovascular Edge-to-Edge Repair (EVEREST II) trial, transcatheter mitral valve repair (TMVR) using MitraClip was compared to mitral valve surgery. Amongst 75 sMR patients, TMVR was equivalent to surgery with respect to the composite primary end-point of freedom from death, surgery for mitral valve dysfunction and ≥ grade 3+ MR after 1 year (82).

Patient selection is one of the most important factors determining success of TMVR as evidenced by the contrasting outcomes of the COAPT (7) and MITRA-FR studies (83) (Table 3).

**Table 3:** Differences between COAPT and MITRA-FR study populations.

**Differences between COAPT and MITRA-FR**		
	**COAPT**	**MITRA-FR**
Number of patients	614	304
Centers	North America	France
Study design	MitraClip + GDMT vs. GDMT alone	MitraClip + GDMT vs. GDMT alone
Outcome	Significant reduction in HF hospitalization, all-cause mortality and cardiovascular death in MitraClip arm	No difference in all-cause death + HF hospitalization at 12 m
EROA, mm^2^ (mean ± SD)	41 ± 15	31 ± 10
> 40 mm^2^	41%	16%
30–40 mm^2^	46%	32%
<30 mm^2^	14%	52%
LVEF, % (mean ± SD)	31 ± 9	33 ± 7
LVEDV, ml/m^2^ (mean ± SD)	101 ± 34	135 ± 35
Procedural complications	8.5%	14.6%
MR Grade ≥ 3+ at 12 m	5%	17%

*COAPT, Cardiovascular Outcomes Assessment of the MitraClip Percutaneous Therapy for Heart Failure Patients with Functional Mitral Regurgitation; MITRA-FR, Percutaneous Repair or Medical Treatment for Secondary Mitral Regurgitation; GDMT, Guideline Directed Medical Therapy; EROA, Effective Regurgitant Orifice Area; LVEF, Left Ventricular Ejection Fraction; LVEDV, Left Ventricular End Diastolic Volume; MR, Mitral Regurgitation; SD, Standard Deviation*.

Generally, those patients with a larger EROA, larger mitral valve area (> 4 cm^2^), and smaller transmitral pressure gradient (< 4 mmHg) have better outcomes following TMVR (84). Worse outcomes are seen in patients with higher RV systolic pressure (RVSP) (85), larger indexed left atrial volume (cut-off 67 ml/m^2^) (86) and greater post-procedure sMR (87).

Pre-procedural assessment by CT is expected to increase with growth of TMVR. The major advantages of CT are accurate measurement of mitral annular dimensions, which has implications for device sizing, landing zone, determining risk of LV outflow tract (LVOT) obstruction, and delineation of mitral annular calcification, in whom TMVR carries higher risk (88). 3D echo accomplishes these with similar accuracy.

Intraprocedural 3D TOE is essential during all steps of TMVR and is superior to 2D TOE (89). Post-procedure, assessment of residual regurgitation can be performed using 3D color doppler. In sMR patients, a post-procedure VCA reduction of > 50% is associated with significant reduction in LA and LV volumes at 6 month follow up (90).

### Summary Recommendations

There are multiple validated pre-operative characteristics on echo identifying likely outcome from surgical repair in sMR;

- Echo and CT both have an important role in the pre-procedure work up for TMVR;
- Intraprocedural 3D TOE is critical to the success of TMVR and superior to 2D TOE.

## Future Directions—Defining the Target Population

The conflicting MITRA-FR and COAPT studies have re-ignited debate regarding patient selection in sMR. At the forefront of this discussion are the imaging parameters used to assess the severity of MR, LV size, and function and their relationship to one another. Several have suggested that this is a consequence of population heterogeneity, with two cohorts: those in whom chronic heart failure and global LV remodeling is the primary pathology and in whom there is a linear relationship between LV dilatation and EROA for any given LVEF. Accordingly, the degree of sMR is “proportionate” to the LV remodeling (with a lower RVol), and these patients are most likely to benefit from heart failure therapies. The contrasting cohort are those with predominant MV valve abnormalities, in whom there is greater regurgitation which is “disproportionate” to LV remodeling. These patients are expected to respond more favorably to mitral valve intervention (91–94). In support of this, subgroup analysis of COAPT patients revealed that the hazard ratio of effect of transcatheter repair crosses 1 as EROA/EDV decreases (95). Others have argued that it is chiefly the haemodynamic burden of sMR that determines benefit from percutaneous mitral intervention, and that RVol and regurgitant fraction (RF) may be more accurate measures (96). The problem with either of these arguments is the ability of echo to measure ventricular volumes, RVol or RF with sufficient accuracy, and future work is needed to explore the potential role of CMR in this regard.

## Conclusion

sMR is common and mortality is high with and without treatment. Medical and surgical therapies do not significantly impact survival but TMVR may reduce mortality and heart failure hospitalization. Assessment of the sMR jet and MV apparatus by 2D and 3D TTE and TOE using a multiparametric approach remain the foundation for assessment. There are however limitations and CMR has the potential to revolutionize the assessment of patients with sMR but is currently underutilized in this field due to lack of research. The role of CT is growing in sMR due to pre-procedural assessment of the patient under consideration for TMVR.

## Author Contributions

All authors contributed to the writing and proofreading of the article.

## Conflict of Interest

The authors declare that the research was conducted in the absence of any commercial or financial relationships that could be construed as a potential conflict of interest. The handling editor declared a past co-authorship with the authors RS and SM.
